# The caries-arresting effect of incorporating functionalized tricalcium phosphate into fluoride varnish applied following application of silver nitrate solution in preschool children: study protocol for a randomized, double-blind clinical trial

**DOI:** 10.1186/s13063-018-2741-1

**Published:** 2018-07-04

**Authors:** Kitty Jieyi Chen, Sherry Shiqian Gao, Duangporn Duangthip, Edward Chin Man Lo, Chun Hung Chu

**Affiliations:** Faculty of Dentistry, The University of Hong Kong, Hong Kong SAR, Pokfulam China

**Keywords:** Dental caries, Children, Fluoride varnish, Silver nitrate, Functionalized tricalcium phosphate

## Abstract

**Background:**

Dental caries in primary teeth is prevalent, affecting millions of children around the world. Functionalized tricalcium phosphate (fTCP) has been incorporated into sodium fluoride (NaF) varnish to enhance the remineralization process. NaF varnish with the adjunctive application of silver nitrate (AgNO_3_) solution is effective in arresting dentine caries. So far, there is no published randomized clinical trial investigating the effectiveness of the adoption of AgNO_3_ solution and NaF varnish containing fTCP in arresting dentine caries in preschool children. The objective of this study is to compare the effectiveness of a 25% AgNO_3_ solution plus a 5% NaF varnish containing fTCP and a 25% AgNO_3_ solution plus a 5% NaF varnish in arresting coronal dentine caries among preschool children when applied semi-annually over a 30-month period.

**Methods/design:**

This is a randomized, double-blind controlled trial. The null hypothesis tested is that no difference exists between the effectiveness of a 25% AgNO_3_ solution plus a 5% NaF varnish with fTCP and a 25% AgNO_3_ solution plus a 5% NaF varnish in arresting dentine caries in preschool children when applied semi-annually. According to the sample size calculation, approximately 2000 3- to 4-year-old kindergarten children will be screened, and at least 408 children with coronal dentine caries will be recruited. The children will be randomly allocated to two treatment groups via stratified randomization: group A – biannual application of a 25% AgNO_3_ solution followed by a 5% NaF varnish, and group B – biannual application of a 25% AgNO_3_ solution followed by a 5% NaF varnish with fTCP. Clinical examinations will be conducted every 6 months to assess whether the carious lesions have become arrested (primary outcome). Confounding factors, such as demographic background and oral hygiene behaviors, will be collected through a parental questionnaire.

**Discussion:**

The effectiveness of the topical application of a 25% AgNO_3_ solution followed by a 5% NaF varnish with fTCP in arresting coronal dentine caries among preschool children remains unknown. Because the proposed caries-arresting methods are simple, noninvasive and low cost, these can be widely recommended for caries control in young children.

**Trial registration:**

ClinicalTrials.gov (U.S.): NCT03423797 on 6 February 2018.

**Electronic supplementary material:**

The online version of this article (10.1186/s13063-018-2741-1) contains supplementary material, which is available to authorized users.

## Background

Dental caries in primary teeth is a common chronic disease in childhood. It has been ranked as the 12th most prevalent condition, affecting around 560 million children worldwide [1]. In Southeast Asian countries, caries prevalence in preschool children is very high (79%) [2], and around half (57%) of Hong Kong’s preschool children have dental caries [3]. These affected children may suffer from tooth pain and infection, possibly resulting in a serious systemic infection with severe complications [4, 5]. Poor dentition also affects children’s growth, development and general health [6]. Furthermore, dental caries has been reported as a common cause of school absenteeism in preschool children [7]. Unfortunately, children from families with lower household incomes who have limited access to dental care have higher caries prevalence and severity [3]. In this context, conventional restorative treatments are often costly and unaffordable for these underprivileged children. In addition, it is a challenge for young children to tolerate the lengthy and complicated procedures of restorative treatment. Therefore, a noninvasive and unsophisticated treatment is required to tackle the burden of dental caries in young children. This study will adopted a non-surgical strategy to arrest dentine caries in primary teeth of preschool children.

### Sodium fluoride (NaF) varnish with functionalized tricalcium phosphate (fTCP)

Results of systematic reviews have suggested that the progression of dental caries could be slowed down or halted without operative procedures [8]. The application of various topical fluoride agents has been reported to be effective in managing dental caries [9]. Fluoride varnish is one of the most commonly applied topical fluoride agents. It can adhere to tooth surfaces for longer times and acts as slow-releasing reservoirs of fluoride. A Cochrane review concluded that NaF varnish had a substantial caries-inhibiting effect on primary teeth with an estimated 37% of pooled caries being prevented [10]. Another review also found that 5% NaF varnish is effective in remineralizing early enamel caries [11].

To enhance the remineralization process, fTCP has been recently incorporated into NaF varnish. fTCP is a novel calcium phosphate system [12]. Calcium and phosphate ions are the primary minerals of the tooth structure. Adequate quantities of both ions must be present to promote ion deposition in a medium during the remineralization process [13]. fTCP works synergistically with fluoride and controls the delivery of calcium and phosphate ions to the tooth substrate. Several laboratory studies on fluoride containing fTCP showed promising results in improving fluoride’s activity and enhancing the remineralization of carious lesions [14–16]. Recently, a study found that silver nitrate (AgNO_3_) and NaF with fTCP reduced the damage of dentine caries by cariogenic biofilm [17]. Although laboratory studies revealed favorable results of incorporating fTCP with NaF varnish for caries management, the question remains as to whether NaF containing fTCP is clinically superior to NaF varnish alone.

Despite its caries-preventive effect, NaF varnish is ineffective at arresting coronal dentine caries [18]. Adjunctive application of AgNO_3_ solution followed by application of NaF varnish has been advocated to improve caries-arrest effectiveness.

### Adjunctive application of AgNO_3_ solution and NaF varnish

AgNO_3_ solution has been used as an antimicrobial agent for years. However, the adoption of AgNO_3_ solution diminished because of the discovery of fluoride for caries management in the mid-nineteenth century [19]. Because AgNO_3_ solution has strong antibacterial properties and NaF varnish has remineralizing properties, the application of AgNO_3_ solution followed by NaF varnish was reintroduced to manage caries in young children [20]. A retrospective study conducted in the United States reported that more than 5000 children were treated with this protocol, and the results revealed that almost all cavitated dentine lesions became arrested after treatment [20]. Subsequently, the number of young children requiring full-mouth rehabilitation under general anesthesia decreased substantially. An in vitro study also confirmed the caries-arresting effect of a 25% AgNO_3_ solution followed by a 5% NaF varnish in remineralizing artificial dentine caries and inhibiting the degradation of collagen [21]. A 12-month randomized clinical trial showed no difference between silver diamine fluoride (SDF) and 25% AgNO_3_ solution followed by a 5% NaF varnish in arresting dentine caries among preschool children [22].

So far, there is no published randomized clinical trial investigating the effectiveness of the adoption of AgNO_3_ solution and NaF varnish containing fTCP in arresting dentine caries in preschool children.

### Objective

The objective of this randomized, double-blind controlled trial is to compare the caries-arresting effectiveness of a 5% NaF varnish with fTCP and that of a 5% NaF without fTCP when applied semi-annually following the application of a 25% AgNO_3_ solution in preschool children over 30 months. The secondary objective is to compare the effectiveness of a 5% NaF varnish with fTCP and that of a 5% NaF without fTCP in preventing dental caries in preschool children over 30 months.

### Hypothesis

The null hypothesis is that there is no difference between the caries-arresting effectiveness of the semi-annual application of a 5% NaF varnish with fTCP and that of a 5% NaF without fTCP following the application of a 25% AgNO_3_ solution in preschool children over 30 months.

## Methods/design

### Trial design

**Fig. 1:**
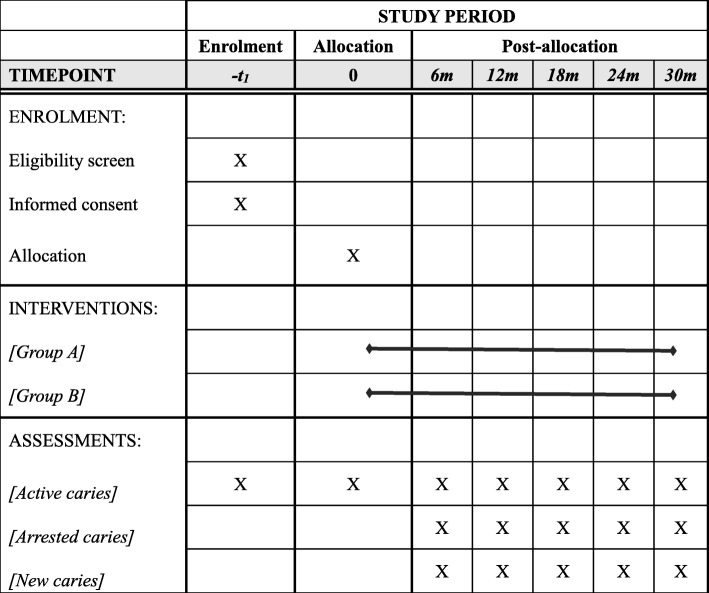
Schedule of enrollment, interventions, and assessments

This is a double-blind, two-armed, parallel-design randomized controlled trial. The Standard Protocol Items: Recommendations for Interventional Trials (SPIRIT) 2013 Statement will be followed in this trial (Additional file 1) [23]. The schedule of this study is presented in Fig. 1.

### Setting

Kindergartens that have joined the outreach dental service provided by Faculty of Dentistry, The University of Hong Kong, will be invited to join this study. Preschool children, who are aged 3 to 4 years, will be invited to participate in the trial. An invitation letter explaining the purposes, procedures of the study and potential adverse effects will be distributed to the parents or legal guardians of the invited children. Written parental consent will be sought prior to the oral examination and dental treatment (Additional file 2).

### Participants

The eligibility criteria for participating children are as follows: (1) be generally healthy, (2) have written parental consent and (3) have at least one cavitated dentine carious lesion. The exclusion criteria are as follows: (1) children who are uncooperative or who refuse examination and (2) children who have major systemic illnesses or are on long-term medications. A tooth with pulpal exposure or with any signs and symptoms of irreversible pulpitis or a non-vital pulp, such as tooth discoloration, will be excluded.

### Screening and recruitment

Oral examination and treatment will take place in kindergartens during school hours. An outreach dental team is comprised of dentists and research assistants. Prior to conducting the study, they will be trained by experienced specialists (ECML and CCH). A calibrated examiner will screen all children who have had parental consent given. After that, participants who meet the inclusion criteria will be recruited.

### Clinical examination

Clinical examination of the children will be performed mainly through a careful visual inspection with the aid of a World Health Organization CPI probe (405/WHO probe, Otto Leibinger, Mühlheim, Germany) and a disposable dental mirror with an attached light-emitting diode for intra-oral illumination (MirrorLite, Kudos Crown Limited, Hong Kong Special Administrative Region of the People’s Republic of China). Tooth status (decayed, missing and filled tooth surfaces (dmfs) scores), tooth mobility and tooth discoloration will be recorded. Caries will be diagnosed at the cavitation level. The visual-tactile assessment will be adopted to assess the status of carious lesions. They will be gently explored with a CPI probe in the centre of the lesion. Great care will be taken to avoid damaging the tooth. For the posterior teeth, all five surfaces (buccal, lingual, mesial, distal and occlusal) will be assessed, while four surfaces (buccal, lingual, mesial and distal) of the anterior teeth will be examined.

The oral hygiene status will be measured using the Visible Plaque Index (VPI). The buccal and lingual surfaces of six index teeth (55, 51, 63, 71, 75 and 83) will be examined. The presence or absence of visible plaque on the carious surface will also be recorded. No dental radiograph will be taken because this study will be conducted in a school-based setting.

A trained dentist will perform the examinations (baseline and follow-ups) throughout the 30-month study. The intra-examiner agreement on the plaque and caries assessment will be performed in 10% of the children at each stage of the study.

### Questionnaire survey

A parental questionnaire regarding each child’s oral hygiene, snacking habits, use of fluoride agents, dental-visit behavior, parental educational level and family monthly income will be administered at baseline and at 12-, 18- and 30-month follow-up visits. To assess the children’s oral-health-related quality of life, the translated and validated Chinese-language Early Childhood Oral Health Impact Scale (ECOHIS) will be adopted in the parental questionnaire. The questionnaires will also assess parental satisfaction with their child’s oral health and dental esthetics. In the follow-up questionnaire, parents of the study children will also be asked to report the post complications of assigned treatments, such as pain, from a treated tooth and gingival irritation around a treated tooth.

### Randomization, treatment allocation and allocation concealment

Participating children with dental caries will be categorized into two strata by the number of carious surfaces they have (one to three or more than three tooth surfaces). The children will be then allocated by a stratified randomization method with a block size of 6 into two groups. The allocation sequence will be generated by a technician who will not be involved in the oral examination and patient allocation. A pile of sealed opaque envelopes with the allocation numbers will be prepared. Those envelopes will be held by a dental assistant in the field. The assistant will know the treatment allocation only when they open the envelope.

### Blinding

Application of the assigned treatment will be carried out after the oral examination. Children’s compliance to fluoride treatment will be recorded and monitored at each treatment visit by an assistant. A double-blinding arrangement will be conducted in this clinical trial. One examiner will perform all the clinical examinations during the study period. The examiner will be blinded to the treatment allocation. The children and their parents also will not be informed of the treatment assignment. The solution and varnish will be applied by an independent operator after the oral examination.

### Interventions

A dentist who is not involved in the assessment of the carious lesions will apply the solution and varnish with a micro-brush on the carious lesion according to the assigned treatment group after the oral examination.

Two intervention groups in this study are as follows:

- Group A: a 25% AgNO_3_ solution (25% silver nitrate, Gordon Labs, Carson, CA, USA) followed by a 5% NaF varnish (Nupro White Varnish, Dentsply, York, PA, USA),
- Group B: a 25% AgNO_3_ solution (25% silver nitrate, Gordon Labs, Carson, CA, USA) followed by a 5% NaF varnish with fTCP (Clinpro White Varnish, 3 M, St. Paul, MN, USA).

After applying the solution and varnish on the carious lesion(s) for arrest purposes, the assigned 5% NaF varnish (0.25 ml) will be applied on all tooth surfaces of each child to prevent dental caries according to their assigned treatment group. After the treatment, the children will be instructed not to eat or drink for 30 min. The intervention will be provided every 6 months.

An individual report on the child’ s oral health status will be distributed to the parents or guardians with a note asking them to report to the study dentist if there are any adverse effects on the treated tooth and/or the surrounding gum and mucosa after treatment. A referral letter will be provided to seek the appropriate dental treatment if needed. Parents and teachers will be informed that if the treatment has successfully arrested the carious lesions, yellow and soft carious surfaces will become hard and black in color.

### Follow-up evaluation

Follow-up clinical examinations will be conducted semi-annually by the same examiner in kindergartens for 30 months. The same diagnostic criteria, equipment and clinical procedures that were used in the baseline examination will be adopted in the follow-up evaluations. The caries status (dmfs index) and oral health status (VPI) of the children will be recorded. In addition, the status of the carious surfaces included in the study at baseline examination and the presence of visible plaque on those carious surfaces will be assessed. Newly developed carious lesions will be recorded and treated with the assigned treatment intervention. The examiner will also look for related side/adverse effects, including blackening of the treated carious surfaces, discolored teeth, tooth mobility and abscess formation during the follow-up oral examinations. Reasons for tooth loss prior to follow-up will be recorded.

### Outcome measure

Outcome measures will be collected at baseline and semi-annually thereafter for 30 months. The primary outcome measure of this study is the proportion of soft (active) carious surfaces that become hardened (arrested). Lesion activity will be assessed based on visual and tactile sensation. A lesion will be recorded as active if softness is detected upon gentle probing, and it will be classified as arrested caries if the lesion is hard [19, 24].

The secondary outcome measure is the caries increment or the change in the dmfs score over time, which will be measured by a calibrated dental examiner who is masked to the group assignment. Visual and tactile inspection without x-rays will be adopted.

### Sample size and power calculation

The results of previous clinical trials showed that around 70% of the active dentine caries became arrested after 30 months [18]. The anticipated caries-arresting rates of group B would be 80%. An absolute difference of 10% for the caries-arresting rates between treatment groups is considered to be clinically significant. The sample size calculation is based on the expected proportion of caries that become arrested, with the power of the study set at 90% (*β* = 0.1) and a two-sided 95% confidence interval (*α* = 0.05) as the statistical significance level by using the G*Power Version 3.1.9.2 (Franz Faul, Universität Kiel, Germany). Therefore, at least 820 active carious tooth surfaces need to be included, or 410 in each study group. Based on the results of epidemiological surveys, we anticipate that the mean number of active caries tooth surfaces of 3-year-old children in Hong Kong is approximately three [25]. The intraclass correlation coefficient for dental caries data at the surface level within the individual would be approximately 0.13 [26]. Following the equation for the required sample size in a multilevel study [27], the design effect will be 1.26. Thus, the estimated sample size would be at least 346 children, or 173 per group. The anticipated dropout rate is 15% [24]; thus, 408 children (204 children in each group) will be needed at baseline.

### Statistical methods

The collected data will be entered into an Excel file by two persons separately, and the data will be proofread to minimize data-entry error. Data will be analyzed using the software SPSS 24.0 for Windows (SPSS Inc., Chicago, IL, USA). The statistical significance level for all tests will be set at 0.05.

An intra-examiner agreement in the diagnosis of dental caries will be assessed by Cohen’s Kappa statistics. An intention-to-treat analysis will be undertaken. The dropout rates of the two groups will be expected to be similar as the treatment protocols and treatment outcomes (arrested caries with blackening of the lesion) are similar.

### Subject-level analysis

A chi-square test and *t* test will be performed, as appropriate, to analyze the differences between the groups regarding the children’s demographic background, oral hygiene and baseline caries experience (dmfs score). A chi-square test will also be conducted to study the difference in dropout rates between groups during the follow-ups. A paired *t* test will be used to compare the changes in ECOHIS, while the McNemar test will be used to compare the changes in oral-health-related behaviors at baseline and follow-up examinations between the different groups. Although the distribution of data for the primary outcome may not be normal, making use of the central limit theorem with the relatively large sample size in this research, the distribution of the mean will be considered normal.

### Surface-level analysis

Because more than one carious lesion may be included from one child, these lesions will possibly be correlated or clustered. Therefore, an adjustment of the clustering effect will be required. Multilevel logistic regression analysis will be adopted to assess the relative effectiveness of a 25% AgNO_3_ and a 5% NaF varnish with or without fTCP in arresting dentine caries. A two-level model will be used: level 1, tooth surface level, and level 2, subject level. Multilevel logistic regression models with a clustering effect adjustment will be performed to analyze the effect of independent variables on the caries-arresting outcomes at the 12-, 18- and 30-month follow-ups. Besides treatment group assignment, other potential variables, such as demographic characteristics (sex and age), oral hygiene status, oral-health-related behaviors, level of parental education, family income, caries condition (baseline dmfs scores, tooth position, lesion site and presence of plaque on lesion), also will be included in the model as covariates.

The secondary outcome (preventive effect) is the mean of caries increment at the 6-, 12-, 18-, 24- and 30-month examinations. A paired *t* test will be performed to assess the difference in the caries increment between the two groups at all follow-up examinations. Multilevel generalized linear models will be adopted to assess the effect of independent variables on the caries increment between two groups at 12, 18 and 30 months.

### Ethical consideration

Ethics approval was obtained from the Institutional Review Board of The University of Hong Kong/Hospital Authority Hong Kong West Cluster (HKU/HAHKWIRB) (IRB reference number: UW17–176). An information sheet about this study will be distributed to parents or legal guardians, and parental consent will be obtained prior to a child’s participation. Children and their parents/legal guardians maintain their rights to receive dental treatment by other dentists. From our previous experiences in conducting clinical trials [11, 18], most participants did not seek help from other dental personnel. In the present study, a question about the receipt of other dental treatments will be included in the baseline and follow-up questionnaires. Medical record notes of the participants may be checked by responsible individuals from the Faculty of Dentistry, The University of Hong Kong or HKU/HAHKWIRB.

## Discussion

This is a randomized, double-blind clinical trial aiming to investigate the caries-arresting effectiveness of the semi-annual application of a 25% AgNO_3_ solution followed by a 5% NaF varnish with/without fTCP among preschool children. The caries-preventive effect, parental satisfaction, changes in oral-health-related quality of life of the study children and the adverse effects of the two intervention groups also will be evaluated.

Due to the limitations of the study, the use of dental radiographs is unavailable and unsafe when conducting a trial in kindergartens [18, 24]. The visual-tactile inspection without x-rays will be adopted for assessing the lesion activity at baseline and follow-up examinations, which is similarly to other caries-arresting studies [28]. To generalize the research findings to general populations, the recruited children will not be asked to change their routine oral care practices including the use of fluoride toothpaste and tooth-brushing habits. Instead, the data of oral care practice habits will be collected by questionnaires and analyzed as confounding factors. The 30-month study period is optimal to investigate the effect of caries-arresting treatment in the long run. The follow-up examinations, which will be conducted every 6 months, will provide the trends or changes of lesion activities over 30 months after receiving the intervention. After finishing this study, all recruited children will be eligible to attend primary schools, and the decayed teeth can be restored by School Dental Care Service which is provided by the Department of Health for primary school students in Hong Kong.

Although clinical trials have demonstrated that SDF is effective in arresting dentine caries in primary teeth, there was a significant increase of new caries development during the trials, as SDF is only applied on the existing cavitated lesions [24, 29]. However, the application of NaF varnish following SDF application at the same visit may increase the risk of fluoride toxicity. It should be noted that SDF is still unavailable in many countries. An alternative to arrest caries using a 5% NaF varnish applied following a 25% AgNO_3_ solution was developed [22]; however, there is a lack of information regarding the preventive effect. Several laboratory studies have shown the anti-caries properties of NaF containing fTCP [12, 14–17], but the clinical efficacy remains unknown. This study will adopt the commercially available product with fTCP, exclusively from 3 M (Clinpro White Varnish), which contains 5% NaF and fTCP less than 5% by weight. The results of the present study will be novel and will provide information regarding the preventive and arresting effect of the application of NaF varnish with/without fTCP following the application of a 25% AgNO_3_ solution in preschool children.

Because the proposed caries management method in this study is simple, noninvasive and low cost, it can be widely recommended and adopted for caries prevention and caries arrest in young children, children with special needs or those living in disadvantaged communities. The results of this study will be beneficial for clinicians and dental health policy-makers when adopting noninvasive approaches to tackle the burden of early childhood caries.

## Trial status

This randomized clinical trial has been registered in ClinicalTrials.gov (U.S.) with the registration number NCT03423797 (Additional file 3). The recruitment of participating kindergarten children and their parents has been in progress from 14 September 2017.

## Additional files

Additional file 1: Standard Protocol Items: Recommendations for Interventional Trials (SPIRIT) Checklist. (PDF 831 kb)

Additional file 2: Parental Consent. (PDF 183 kb)

Additional file 3: World Health Organization (WHO) Trial Registration Data Set. (PDF 365 kb)

## Abbreviations

HKU/HAHKWIRB: Institutional Review Board of The University of Hong Kong/Hospital Authority Hong Kong West Cluster
AgNO_3_: Silver nitrate
dmfs: Decayed, missing and filled tooth surfaces
ECOHIS: Early Childhood Oral Health Impact Scale
fTCP: Functionalized tricalcium phosphate
IRB: Institutional Review Board
NaF: Sodium fluoride
SDF: Silver diamine fluoride
SPIRIT: Standard Protocol Items: Recommendations for Interventional Trials
VPI: Visible Plaque Index
