# Supplementary material for: The caries-arresting effect of incorporating functionalized tricalcium phosphate into fluoride varnish applied following application of silver nitrate solution in preschool children: study protocol for a randomized, double-blind clinical trial
Source: Trials. 2018 Jul 4;19:352. doi: 10.1186/s13063-018-2741-1 (PMC6032524; doi:10.1186/s13063-018-2741-1)
Supplement: Supplementary file 2 — Parental Consent. (PDF 183 kb) [file 13063_2018_2741_MOESM2_ESM.pdf]

K1 Only

Faculty of Dentistry
The University of Hong Kong

Re: The caries-arresting effect of incorporating fuctionalized tricalcium phosphate to fluoride varnish applied following silver nitrate solution in preschool children

01 Oct 2017
Dear parents/guardians:

Your child is being invited to take part in a research study. Before you decide, it is important for you to understand why the research is being done and what it will involve. Please take time to read the following information carefully and discuss it with friends, relatives and your family doctor if you wish. Ask us if there is anything that is not clear or if you would like more information. Take time to decide whether or not you wish your child to take part.

Faculty of Dentistry of The University of Hong Kong is now conducting a clinical study among preschool children. The aim of this study is to compare the effectiveness of 25% silver nitrate ($AgNO_3$) and sodium fluoride (NaF) with or without functionalized tricalcium phosphate (fTCP) in arresting tooth decay in the primary teeth. Approximately, 558 children with decayed tooth will be invited to join. Studies showed 5% NaF varnish was effective in preventing tooth decay. To enhance the effectiveness, 25% $AgNO_3$ solution was proposed to use in combination with NaF varnish. Studies found NaF varnish containing fTCP more effective in remimeralization. There is no serious side effect reported in the literature. The successfully arrested carious lesion will turn black and hard; and this is a common outcome for successful treatment. Please note that there will be no dark staining on sound teeth.

The details of the activity would be as follows:

- Target: Pre-school children of 3-4 years of age (K1 students);
- Proposed period: 30-month caries preventive treatment; Oral examination and intervention will be provided biannually;
- Activities: (1) oral health education; (2) parental questionnaire survey; (3) oral examination; (4) silver nitrate plus sodium fluoride with/without functionalized tricalcium phosphate treatment.

These activities will be carried out in the kindergarten. If your child has tooth decay, he/she will be randomly allocated to receive one dental treatment either group A (AgNO3 plus NaF) or group B (AgNO3 plus NaF plus fTCP) treatment. The potential risks for this study include: 1) You child may feel discomfort during the examination; 2) Gum and mucosa may have transient irritation. During the dental examination, no radiography will be taken. Each participating child will receive a report on the oral health. Children who need any other dental services such as extraction can be treated by a dentist at your own cost.

It is up to you to decide whether or not to let your child to take part. If you decide to take part in the study, please fill in the questionnaire attached and authorize us to provide a dental examination to your children in school time. The oral examination and treatment will take for 1 to 2 minutes. No lifestyle or dietary restriction is required during the study period. You are still free to withdraw at any time and without giving a reason. This will not affect the standard of care you receive in the future.

You have the rights of access to personal data and publicly available study results, if and when needed. Under the laws of Hong Kong (in particular the Personal Data (Privacy) Ordinance, Cap 486), you enjoy or may enjoy rights for the protection of the confidentiality of your personal data, such as those regarding the collection, custody, retention, management, control, use (including analysis or comparison), transfer in or out of Hong Kong, non-disclosure, erasure and/or in any way dealing with or disposing of any of your personal data in or for this study. For any query, you should consult the Privacy Commissioner for Privacy Data or his office (Tel No. 2827 2827) as to the proper monitoring or supervision of your personal data protection so that your full awareness and understanding of the significance of compliance with the law governing privacy data is assured.

By consenting to participate in this study, you expressly authorize:

a) the principal investigator and his research team and the ethics committee (Institutional Review Board of the University of Hong Kong / Hospital Authority Hong Kong West Cluster) responsible for overseeing this study to get access to, to use, and to retain your personal data for the purposes and in the manner described in this informed consent process; and
b) the relevant government agencies (e.g. the Hong Kong Department of Health) to get access to your personal data for the purposes of checking and verifying the integrity of study data and assessing compliance with the study protocol and relevant requirements.

It is possible that when taking part in the oral examination, your child may feel discomfort. If your child is too unwilling to be checked, the oral examination will stop immediately. If your child is harmed by taking part in this study, there are no special compensation arrangements. If your child is harmed due to someone's negligence, then you may have grounds for a legal action. Regardless of this, if you wish to complain about any aspect of the way your child has been approached during the course of this study, the normal health service complaints mechanisms may be available to you.

After examination, each of the participants will receive a set of souvenir. We hope that the oral examination will help you to understand the oral health status of your child. This study is self-funded and has been reviewed by Institutional Review Board of the University of Hong Kong / Hospital Authority Hong Kong West Cluster. If you have any enquiry, please contact Prof. Chu Chun Hung at 2859 0287 during office hours. Please complete the following consent if you agree to participate in this study. Thank you very much.

Yours faithfully,

Chu

Prof. Chu Chun Hung
Clinical Professor in Community and Family Dentistry
Faculty of Dentistry, The University of Hong Kong

Faculty of Dentistry
The University of Hong Kong

K1 Only

PATIENT/SUBJECT CONSENT FORM

Title of Project: **Re: The caries-arresting effect of incorporating fuctionalized tricalcium phosphate to fluoride varnish applied following silver nitrate solution in preschool children**

Name of Researcher: Prof. Chu Chun Hung

Please initial box

1. I confirm that I have read and understood the information sheet dated __/__/__ for the above study and have had the opportunity to ask questions. ☐

2. I understand that my participation is voluntary and that I am free to withdraw at any time, without giving any reason, without my medical care or legal rights being affected. ☐

3. I understand that sections of any of my medical notes may be looked at by responsible individuals from Faculty of Dentistry, The University of Hong Kong or from Institutional Review Board of the University of Hong Kong / Hospital Authority Hong Kong West Cluster.
I give permission for these individuals to have access to my records. ☐

4. I agree to take part in the above study. ☐

______________________	__________	______________________
Name of child/Parent or Guardian	Date	Signature
Dr. Chu Chun Hung	2017.10.01	Chu
Researcher	Date	Signature
