# Supplementary material for: The caries-arresting effect of incorporating functionalized tricalcium phosphate into fluoride varnish applied following application of silver nitrate solution in preschool children: study protocol for a randomized, double-blind clinical trial
Source: Trials. 2018 Jul 4;19:352. doi: 10.1186/s13063-018-2741-1 (PMC6032524; doi:10.1186/s13063-018-2741-1)
Supplement: Supplementary file 3 — World Health Organization (WHO) Trial Registration Data Set. (PDF 365 kb) [file 13063_2018_2741_MOESM3_ESM.pdf]

Additional File 3. WHO Trial Registration Data Set

Data category	Information
Primary registry and trial identifying number	ClinicalTrials.gov NCT03423797
Date of registration in primary registry	31st Jan 2018
Secondary identifying numbers	UW 17-176
Source(s) of monetary or material support	Not applicable
Primary sponsor	Not applicable
Contact for public queries	Chu Chun Hung<br>chchu@hku.hku; (+852) 2859 0246
Contact for scientific queries	Chu Chun Hung<br>chchu@hku.hku; (+852) 2859 0246
Public title	The Caries-arresting Effect of Incorporating fTCP to NaF Varnish Applied Following $AgNO_3$ in Preschool Children
Scientific title	Effectiveness of Biannual Application of Silver Nitrate Solution Followed by Sodium Fluoride Varnish With or Without Functionalized Tricalcium Phosphate in Arresting Caries in Preschool Children
Countries of recruitment	Hong Kong SAR, China
Health condition(s) or problem(s) studied	Dental Caries
Intervention(s)	Active Comparator: 25% $AgNO_3$ solution followed by 5% NaF;<br>Experimental: 25% $AgNO_3$ solution followed by 5% NaF with fTCP.
Key inclusion and exclusion criteria	Inclusion criteria: Children have at least one tooth with cavitated dentine carious lesion;<br>Exclusion criteria: Children have severe forms of dental hypoplasia, under dental treatment or wearing orthodontic devices, have major systemic illness, and are on long-term medication
Study type	Interventional<br>Allocation: randomized<br>Intervention model: parallel assignment<br>Masking: double blind Primary purpose: treatment<br>Phase III
Date of first enrolment	14 Sep 2017
Target sample size	408 children
Recruitment status	Recruiting

Primary outcome(s)	The effectiveness of adjunctive application of 25% AgNO3 solution and 5% NaF varnish with or without fTCP in arresting dentine caries of primary teeth in preschool children over 30 months
Key secondary outcomes	Compare the effectiveness of 5% NaF varnish with fTCP and that of 5% NaF without fTCP in preventing dental caries in preschool children over 30 months
